# Brazil was certified by the World Health Organization for having eliminated lymphatic filariasis: what now?

**DOI:** 10.1186/s13071-025-06707-0

**Published:** 2025-03-31

**Authors:** Eduardo Brandão, Paula Oliveira, Maria Almerice Lopes da Silva, Abraham Rocha

**Affiliations:** National Reference Service for Filariasis, Aggeu Magalhães Institute, Oswaldo Cruz Foundation, Recife, Brazil

**Keywords:** Lymphatic filariasis, Elimination, Epidemiological surveillance

## Abstract

Lymphatic filariasis (LF) is a debilitating and stigmatizing disease. In Brazil, the disease has been the target of control and elimination strategies for more than 25 years. Recently, the country received the certificate of elimination of LF as a public health problem. In this post-elimination moment, the question arises: what now? Given the possibility of emergence and reemergence of the disease, it is important that epidemiological surveillance measures are reviewed and applied effectively.

**Graphical Abstract:**

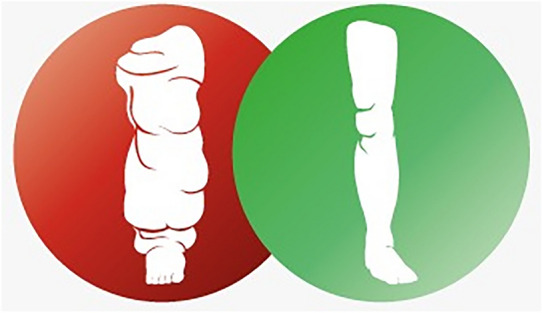

## Correspondence

Lymphatic filariasis (LF) is a chronic parasitic disease primarily transmitted by vectors of the genera *Anopheles*, *Aedes*, and *Culex*, which still threatens 657 million individuals in 39 countries in tropical and subtropical regions of the world. The etiological agents are helminths of the species *Brugia malayi*, *Brugia timori*, and *Wuchereria bancrofti*, the latter being responsible for approximately 90% of cases. Infection usually occurs in childhood, and in most cases it is asymptomatic; however, it is in adulthood that the disabling forms of the disease appear. LF gained notoriety for its most stigmatizing and debilitating clinical presentation, lower limb lymphedema, also known as elephantiasis [1].

Owing to the biopsychosocial and economic impacts caused by the disease, LF has come to be considered a parasitic disease of significant relevance, and it has become one of the major concerns of the main global health bodies. In 1997, the World Health Organization (WHO) understood that, although the species *Brugia malayi* has several felids and primates as a reservoir, humans represent the main host of LF; they also understood that it has specific treatment. In this context, LF was included in the group of eradicable diseases; the Global Program to Eliminate Lymphatic Filariasis (GPELF) was created, which had the initial goal of eliminating the disease from the world as a public health problem by 2020 [2], with this goal being postponed to 2030 [3].

Signatory to the GPELF, Brazil launched the National Plan for the Elimination of LF, which followed the same pillars recommended by the WHO: interrupting transmission with antifilarial drugs and providing assistance to those with morbidity [4]. After more than 25 years of actions that included treatment administration, surveillance at sentinel sites, vector control, and assistance to those with morbidity, especially in the most affected areas, much progress has been made, especially with regard to the transmission of the disease in the remaining foci in Brazil, where the last case reported in an endemic area was in 2017 in Recife, in the State of Pernambuco. Recently, on 30 September 2024, these efforts led the country to receive the WHO certificate of elimination of LF as a public health problem [5].

The news that the disease has been eliminated raises important questions, such as: Have all the locations where there were positive cases been adequately managed? Will new cases of the disease appear? Can we dismantle the actions? What surveillance model should we follow? How can we continue to identify unassisted carriers of filarial morbidity?

Understanding the complexity involved in this silent disease involves taking into account several associated factors involved in the dynamics of the disease. These include the lack of basic sanitation, the high population density of vectors, and the intense migratory processes of individuals from endemic countries, the lack of diagnostic tests that quickly and accurately signal the occurrence of new cases in areas with a certificate of elimination, and the existence of a large number of people with lymphedema and hydrocele. Consequently, it can be assumed that LF will remain a topic of discussion for many years. Brazil’s health systems and public health policies must be prepared for the demands that arise at this stage of the program.

Faced with the challenges presented and considering the sustainability of the results achieved in the country, it is extremely important that epidemiological surveillance measures are reviewed and continue to be carried out [3]. These strategies should include preparing informative guidelines related to the diagnosis and treatment of the disease, training a greater number of health professionals capable of identifying suspected cases of the parasitosis, and monitoring of breeding sites of the vector. They should also include conducting xenomonitoring surveys in the areas, monitoring of new cases by performing tests on immigrants from countries considered endemic, and evaluating locations’ bordering areas that were subjected to mass treatment but were not targeted by the elimination plan actions. They should reassess, through epidemiological surveys, the former endemic regions and provide multidisciplinary and interdisciplinary care for those with the disease, including mental health, physiotherapy, and nutrition of these individuals, an aspect that has been neglected for a long time and that directly interferes with the quality of life of these individuals.

Other initiatives, equally important for this moment of GPELF, must include the evaluation of the diagnostic tools currently available in this new situation, as well as the development of new accurate tools capable of guaranteeing the absence of infection, thus reducing the possibility of emergence or reemergence of new foci.

## Data Availability

No datasets were generated or analyzed during the current study.

## Abbreviations

GPELF: Global Program to Eliminate Lymphatic Filariasis
LF: Lymphatic filariasis
WHO: World Health Organization
